# Dynamic profiles of 25 serum biomarkers in acute myocardial infarction

**DOI:** 10.3389/fcvm.2025.1631648

**Published:** 2025-09-25

**Authors:** Xuefeng Ai, Yuanyuan Wang, Mingwei Jiang, Qianyu Han, Hongli Geng, Cheng Wang, Jirui Chen, Lei Xue, Yuxiang Jin

**Affiliations:** ^1^ Department of Thoracic Surgery, Shanghai Changzheng Hospital, Second Affiliated Hospital of Naval Military Medical University, Shanghai, China; ^2^ Department of Thoracic Surgery, Shanghai Pulmonary Hospital, Tongji University School of Medicine, Shanghai, China

**Keywords:** acute myocardial infarction, LAD ligation, serum biomarkers, temporal dynamics, early diagnosis, rat model

## Abstract

**Background:**

Acute myocardial infarction (AMI) is a leading cause of cardiovascular mortality and perioperative complications in the elderly (>65 years). However, existing clinical biomarkers (e.g., troponin) still lack sufficient sensitivity for ultra-early diagnosis. A comprehensive understanding of the dynamic changes in serum biomarkers post-AMI is crucial for developing novel diagnostic strategies.

**Methods:**

A rat AMI model was established by surgical ligation of the left anterior descending (LAD) coronary artery. Cardiac function was evaluated via echocardiography and triphenyltetrazolium chloride (TTC) staining at 24 h post-AMI. Blood samples were collected at baseline (pre-anesthesia) and at 1, 2, 6, 12, 24, and 48 h post-LAD ligation. Serum levels of 25 biomarkers were measured by ELISA, including: α-smooth muscle actin (α-SMA), aminopeptidase N (ANPEP), B-type natriuretic peptide (BNP), C-C chemokine receptor type 2 (CCR2), C-reactive protein (CRP), connective tissue growth factor (CTGF), C-X-C motif chemokine ligand 16 (CXCL16), cystatin C (Cys), dopamine D2 receptor (D2D), glucagon-like peptide-1 (GLP-1), homocysteine (Hcy), chemokine-like factor 1 (CKLF1), high-sensitivity troponin I (hs-TnI), interleukin-1β (IL-1β), interleukin-6 (IL-6), lipoprotein(a) (Lp-α), monocyte chemoattractant protein-1 (MCP-1), matrix metalloproteinase-9 (MMP-9), NOD-like receptor family pyrin domain-containing 3 (NLRP3), plasminogen activator inhibitor-1 (PAI-1), S100 calcium-binding protein A8 (S100A8), solute carrier family 31 member 1 (copper transporter, SLC31A1), tissue inhibitor of metalloproteinases-1 (TIMP-1), tumor necrosis factor-α (TNF-α), and vascular endothelial growth factor A (VEGF-A).

**Results:**

At 24 h post-AMI, LVEF was significantly decreased in the AMI group (63.84 ± 2.48% vs. 38.83 ± 2.62%, *p* < 0.001), with an infarct size of 28.70 ± 1.43%. A total of 25 blood biomarkers potentially associated with AMI were detected. Among them, 17 biomarkers showed rapid elevation within 1 h post-AMI (excluding IL-6, TNF-α, ANPEP, D2D, CXCL16, Lp-α and α-SMA). IL-6, TNF-α and ANPEP exhibited significant elevation at 2 h post-AMI, while CXCL16 showed obvious elevation at 6 h and α-SMA demonstrated significant elevation at 12 h. However, S100A8, GLP-1, MMP-9 and NLRP3 showed a decrease at 2 h, although their overall trend within 48 h was upward. Lp-α and D2D remained below baseline levels throughout the observation period, with both showing levels below baseline at 1 h post-AMI. They returned to baseline levels at 12 h and 2 h respectively, followed by rapid decreases again

**Conclusion:**

This study is the first to systematically characterize the dynamic profiles of 25 serum biomarkers following AMI in rats, revealing that: (1) IL-1β, S100A8, BNP, SLC31A1 and Cys may serve as an ultra-early (1 h) diagnostic panel (increase of over 70% at 1 h); (2) the delayed elevation of α-SMA and CXCL16 may be associated with the initiation of myocardial repair; (3) the suppression of Lp-α and D2D might reflect compensatory protective mechanisms.

## 1 Introduction

Cardiovascular diseases (CVDs) remain one of the leading causes of global mortality and disability, with AMI being a major clinical concern due to its high fatality and disability rates (1). AMI is a pathological process characterized by acute coronary occlusion leading to myocardial ischemia, hypoxia, and ultimately cardiomyocyte necrosis. Although reperfusion therapies (such as thrombolysis and percutaneous coronary intervention) have significantly improved AMI outcomes in recent years, early diagnosis and intervention remain crucial for reducing mortality (2, 3). Currently, clinical diagnosis primarily relies on electrocardiography (ECG) and myocardial injury biomarkers (e.g., troponin I/T, B-type natriuretic peptide) (4–7). However, the sensitivity and specificity of these indicators in the early stages (particularly within 1–2 h) of AMI onset remain limited. Therefore, identifying earlier and more precise serological biomarkers is of great significance for early AMI warning and treatment optimization.

Current clinical diagnosis of AMI primarily relies on myocardial injury biomarkers, with cTnI/cTnT being the gold standard (8, 9). cTnI/cTnT are structural proteins released into the circulation upon necrosis of cardiomyocytes following ischemic injury. Their release kinetics are characterized by a detectable rise within 3–6 h, peaking at 12–24 h, and remaining elevated for 5–10 days after AMI. While the introduction of high-sensitivity cardiac troponin (hs-cTn) (8) assays has significantly improved early detection sensitivity, diagnostic uncertainty may still persist in the hyper-acute phase (<3 h) due to potential overlaps in absolute values and kinetic patterns. The traditional biomarker creatine kinase-MB (CK-MB) (8) exhibits an earlier rise but lower cardiac specificity and a shorter window of elevation (peaking at 10–18 h and lasting 2–3 days), thus often serving as an auxiliary diagnostic tool. These limitations underscore the critical need to explore biomarkers that change during the very early stages of ischemia, even prior to overt necrosis, to achieve true ultra-early diagnosis.

Following myocardial ischemic infarction, cardiomyocyte necrosis triggers complex inflammatory responses (10), oxidative stress (11), and myocardial remodeling processes (12), during which various bioactive molecules are released into the bloodstream. In recent years, with the development of proteomics and molecular biology technologies, an increasing number of potential AMI-related markers have been reported, such as inflammatory factors (IL-6, TNF-α), myocardial fibrosis-related proteins (CTGF, MMP-9), and metabolic regulatory molecules (GLP-1, Hcy) (13, 14). However, the dynamic patterns of these markers and their clinical significance at different stages of AMI have not been fully elucidated. Moreover, most studies have focused on single or a few markers, lacking systematic analysis of the temporal characteristics of continuous changes in multiple indicators after AMI, which limits their application in clinical practice.

Animal models are important tools for studying AMI pathological mechanisms and marker screening. Among them, the rat Left Anterior Descending Coronary Artery (LAD) ligation model is widely used due to its operational stability and high similarity to human AMI pathological changes (15). This study employs this model to systematically detect the dynamic changes of 25potential biomarkers in serum at different time points (0, 1, 2, 6, 12, 24, and 48 h) before and after AMI, including inflammatory factors (IL-1β, IL-6, TNF-α, MCP-1), myocardial injury markers (hs-TnI, BNP), oxidative stress-related molecules (Hcy, S100A8), and vascular remodeling factors (VEGF-A, MMP-9, TIMP-1), This study primarily explores the potential comprehensive systemic response triggered by cardiac injury, aiming to construct a continuous change profile of serum markers after AMI.

The main objectives of this study include: (1) screening markers that are significantly elevated in the early stages of AMI (1–6 h) to provide new evidence for clinical early diagnosis; (2) analyzing the temporal change characteristics of different markers to reveal the dynamic patterns of inflammation, fibrosis, and metabolic regulation after AMI; (3) exploring the potential mechanisms underlying the lack of elevation of certain markers (such as Lp-α and D2D) after AMI. Our findings will not only contribute to a deeper understanding of the pathophysiological processes of AMI but may also provide a more comprehensive biomarker detection strategy for clinical practice, thereby optimizing early diagnosis and individualized treatment of AMI.

## 2 Materials and methods

### 2.1 Establishment of rat AMI model

All animal care and experimental procedures were conducted in accordance with the guidelines of the Animal Care and Use Committee of Shanghai Changzheng Hospital. Male Sprague-Dawley rats (weight: 200–250 g; age: 5 weeks) were provided by Shanghai Jiesijie Laboratory Animal Co., Ltd. The rats were housed in a standard SPF facility under controlled conditions (temperature: 22 ± 2°C; humidity: 50%–60%; 12 h/12 h light/dark cycle) with free access to food and water. After a 1–2 week acclimatization period, the experiments were initiated.

A total of 26 rats were used in this study, distributed as follows: the sham surgery group (Sham, *n* = 8), 4 were allocated for TTC staining experiments; and 4 were used for ELISA assays; and the AMI group (*n* = 18). Among the AMI group, 4 rats were utilized in preliminary pilot experiments; 3 were excluded due to the absence of significant infarction as confirmed by postoperative echocardiography; 1 died during the surgical procedure; 4 were allocated for TTC staining experiments; and 6 were used for ELISA assays. The AMI model was established via LAD ligation, as previously described (16). Briefly, rats were anesthetized with 2% isoflurane (R510-22-10, RWD Life Science), intubated, and connected to a small-animal ventilator (ALC-V8-SLA, Shanghai ALCOTT BIOTECH), and maintained under anesthesia with 1.5% isoflurane. A left thoracotomy was performed between the fourth and fifth ribs to expose the heart. The LAD was ligated with a 6-0 silk suture (EH7297H, Ethicon) 2–3 mm below the left atrial appendage. Successful ligation was confirmed by immediate pallor of the anterior left ventricular wall. The chest was then closed, anesthesia was discontinued, and rats were allowed to recover on a heating pad under close observation. All experimental rats were euthanized by inducing deep anesthesia through isoflurane overdose, followed by cervical dislocation as a secondary physical method to ensure death confirmation.

### 2.2 Blood sample collection post-AMI

Blood samples were collected from the retro-orbital venous plexus of all rats in both the sham and AMI groups at baseline (pre-anesthesia) and at 1, 2, 6, 12, 24, and 48 h after acute myocardial infarction. Prior to collection, topical anesthesia (1% tetracaine) was applied to the eye. Approximately 0.2 ml of whole blood was drawn using heparinized capillary tubes (diameter: 1.0 mm). Hemostasis was achieved with sterile cotton balls, and chloramphenicol eye drops were administered to prevent infection. Blood samples were incubated at room temperature for 30 min, centrifuged at 3000 rpm for 15 min (4°C), and the serum aliquots were diluted to 0.4 ml with ELISA diluent and stored at −80°C.

### 2.3 ELISA assay

Serum levels of 25 biomarkers (α-SMA, ANPEP, BNP, etc.) were quantified using commercial ELISA kits (DY008B, R&D systems) according to the manufacturer's protocols. Briefly, serum samples and standards were added to pre-coated 96-well plates, followed by incubation with detection antibodies (abcam: ab7817, ab108310, ab19645, ab273050, ab259862, ab318148, ab109508, ab85367, ab111125, ab133635, ab180512, ab283818, ab9324, ab208184, ab219045, ab76003, ab263899, ab317604, ab180735, ab317432, ab216432, ab307164, ab100787; Biorbyt: orb1736502, Invitrogen: PA5115068) and enzyme-linked secondary antibodies (ab6721, abcam). Color development was initiated with TMB substrate, and absorbance was measured at 450 nm. All steps were performed in duplicate, with quality controls and standard curves (R^2^ > 0.99). Data were acquired using a microplate reader (BioTek Synergy H1), and statistical analysis was performed using SPSS software (version 20.0), while graphs were generated with GraphPad Prism (version 8.0).

### 2.4 Echocardiography

At 24 h post-AMI, transthoracic echocardiography was performed on all surviving rats using a high-resolution small-animal ultrasound system (VisualSonics Vevo 2,100). Under 1.5% isoflurane anesthesia, rats were placed in a supine position on a temperature-controlled platform. After chest hair removal and application of ultrasound gel, standard left ventricular long- and short-axis views were obtained using an MS250 probe (21 MHz). Left ventricular ejection fraction (LVEF) were measured. All images were acquired by two blinded operators, with three consecutive cardiac cycles averaged per parameter.

### 2.5 TTC staining

At 24 h post-AMI (Including sham group), hearts were rapidly excised under deep anesthesia, rinsed with ice-cold PBS, and frozen at −20°C for 20 min. The hearts were sectioned transversely into 3 mm-thick slices and incubated in 1% TTC (T8170, Solarbio) solution (pH 7.4, 37°C) for 15 min in the dark. Viable myocardium stained brick-red due to dehydrogenase activity, while infarcted areas remained pale. After fixation with 4% paraformaldehyde for 30 min, sections were photographed, and infarct size (% of left ventricle) was quantified using ImageJ.

### 2.6 Statistical analysis

Statistical analysis was performed using SPSS 20.0 (SPSS, Chicago, IL, USA). Normally distributed continuous data were expressed as mean ± standard deviation, and comparisons between two samples were conducted using the *t*-test. Intergroup differences were analyzed by one-way ANOVA with Bonferroni *post hoc* tests (SPSS, Chicago, IL, USA). Two-sided tests were used, with statistical significance set at *P* < 0.05.

## 3 Results

### 3.1 Successful establishment of acute myocardial infarction model

**Figure 1:**
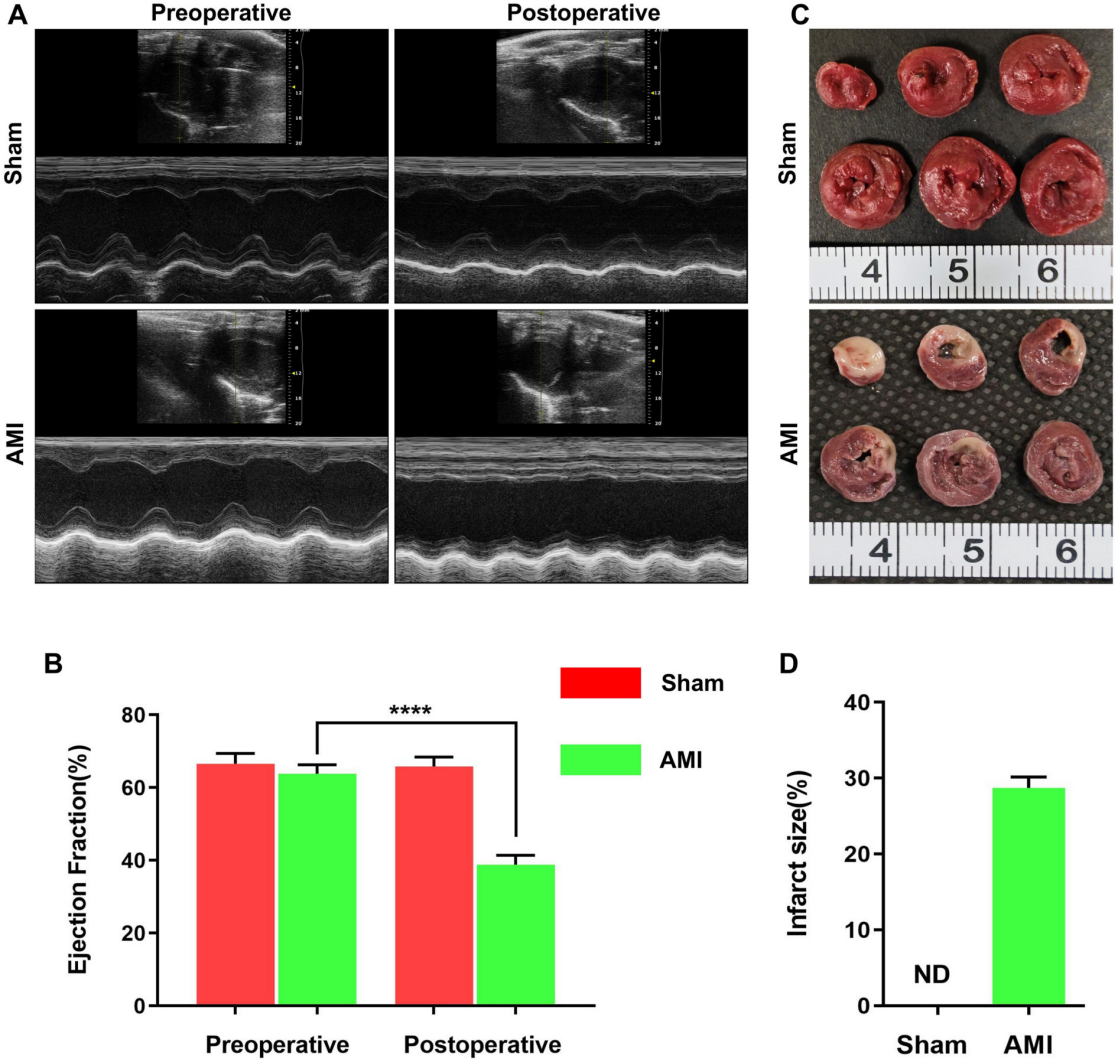
Successful establishment of acute myocardial infarction (AMI) rat model by LAD ligation. **(A)** Representative echocardiographic images from the sham (*n* = 4) and AMI (*n* = 6) groups, illustrating impaired wall motion in the infarct area. **(B)** Quantitative analysis of left ventricular ejection fraction (LVEF) showing significant reduction post-AMI (*P* < 0.0001 vs. pre-operation). **(C)** Representative TTC-stained heart sections from both groups. The pale unstained region indicates the infarcted area in the AMI heart, while the sham group shows no infarction. **(D)** Quantification of myocardial infarct size, confirming extensive infarction in the AMI group (28.70% ± 1.43%) and no infarction (0%) in sham group. Data presented as mean ± SEM.

The AMI model was successfully established by ligating the LAD in rats. Echocardiographic images at 24 h post-AMI showed significantly reduced wall motion in the infarct zone (Figure 1A), while TTC staining revealed distinct pale infarct areas (Figure 1C). In the sham group, TTC staining confirmed the absence of myocardial infarction. Quantitative analysis demonstrated a significant decrease in left ventricular ejection fraction (LVEF) in the AMI group (38.83% ± 2.62% vs. pre-operative 63.84% ± 2.48%, *P* < 0.001), confirming successful LAD ligation (Figure 1B). In contrast, the sham group showed no significant difference in LVEF before (67.59% ± 2.58%) and after (68.85% ± 3.41%) the procedure. TTC staining further verified myocardial infarction in the AMI group with an infarct size of 28.70% ± 1.43% (Figure 1D). These data indicate the stable and reliable establishment of the AMI model, suitable for subsequent dynamic analysis of serum biomarkers.

### 3.2 Serum biomarkers showing sustained elevation within 1 h post-AMI

**Figure 2:**
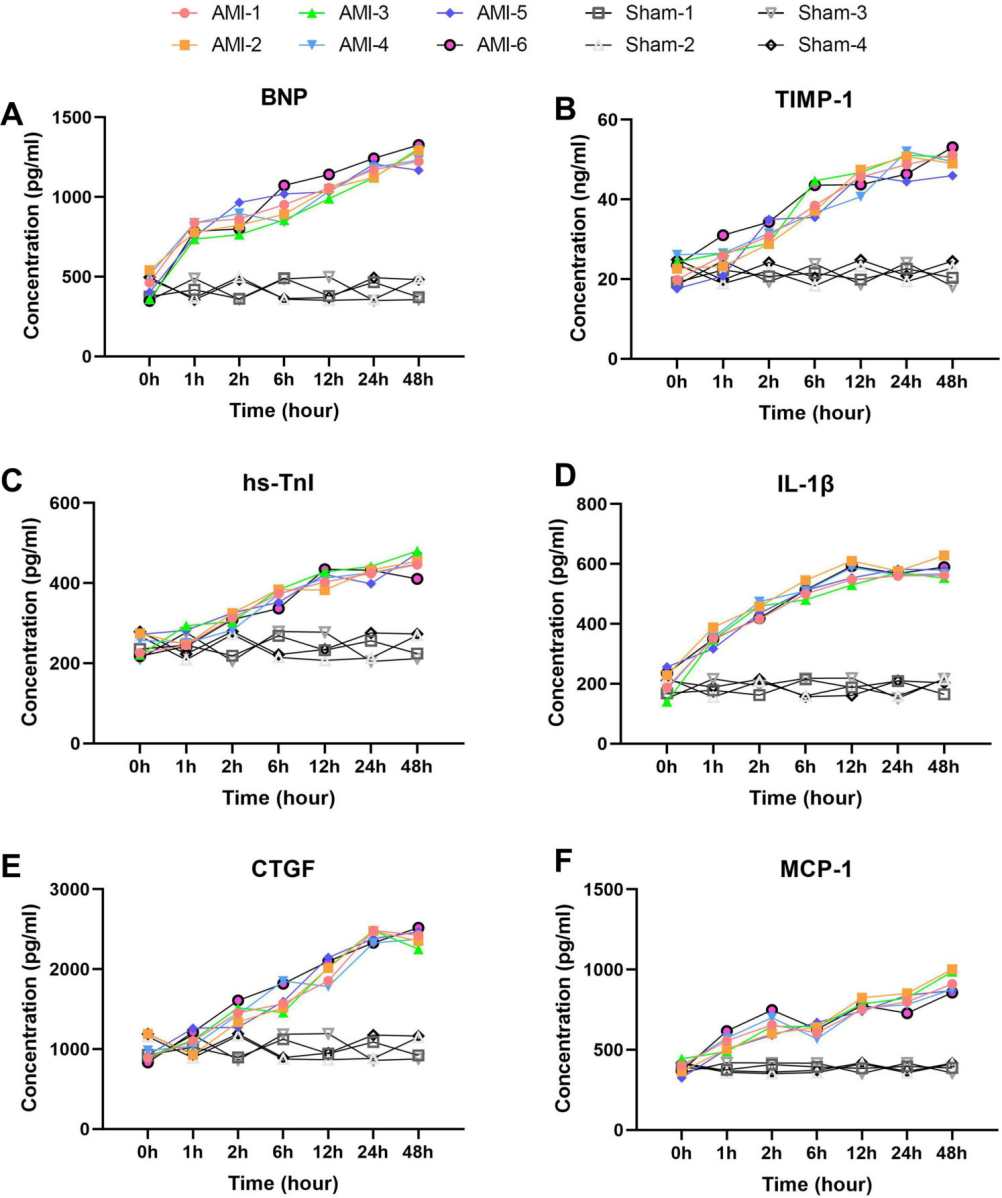
Rapid and sustained elevation of serum biomarkers within 1 h post-AMI. **(A)** BNP levels showing rapid elevation at 1 h (*P* = 4.95 × 10^−5^ vs. baseline) with sustained increase to 48 h (*P* = 0.0004). Sham group showed no significant changes (*P* > 0.05). **(B)** TIMP-1 demonstrating progressive elevation from baseline, reaching peak at 48 h. Levels remained stable in sham group. **(C)** hs-TnI exhibiting gradual increase with first significant change at 2 h (*P* = 0.005). No significant fluctuation was observed in sham group. **(D)** IL-1β displaying rapid response at 1 h (*P* = 0.00091) and peak at 12 h. Sham group maintained baseline levels throughout. **(E)** CTGF showing marked elevation from 2 h (*P* = 0.0036) to 24 h (*P* = 0.0013). Sham animals exhibited no significant changes. **(F)** MCP-1 revealing biphasic pattern with initial peak at 2 h (*P* = 0.00012) and secondary rise at 48 h (*P* = 0.00217). Sham group levels remained unaltered. Data are mean ± SEM, *n* = 6, All *P* values represent comparisons with baseline measurements.

Among myocardial injury markers, BNP and hs-TnI exhibited significant time-dependent increases (Figures 2A,C), while the sham group showed no significant changes across all time points (*P* > 0.05). BNP levels rose sharply from a baseline of 437.57 ± 80.15 pg/ml–787.38 ± 42.95 pg/ml within 1 h (*P* = 4.95 × 10^−5^), peaking at 48 h (1,257.44 ± 60.27 pg/ml, *P* = 0.0004). Notably, levels exceeded 1,000 pg/ml by 12 h (1,051.79 ± 50.27 pg/ml). In contrast, sham group BNP levels remained stable. hs-TnI showed progressive elevation from baseline (245.08 ± 26.47 pg/ml), with the first significant increase at 2 h (310.09 ± 15.95 pg/ml, *P* = 0.005), reaching a peak at 48 h (452.57 ± 24.76 pg/ml, *P* = 0.092). Sham group hs-TnI levels showed no significant fluctuations (*P* > 0.05).

Inflammatory markers IL-1β and MCP-1 displayed distinct dynamic patterns (Figures 2D,F). IL-1β showed rapid response, increasing from 204.86 ± 42.57 pg/ml–351.31 ± 22.83 pg/ml within 1 h (*P* = 0.00091), peaking at 12 h (570.58 ± 31.19 pg/ml) before stabilizing. Sham group IL-1β levels remained within baseline range. MCP-1 exhibited a biphasic pattern: rapid elevation to 540.81 ± 49.78 pg/ml at 1 h (*P* = 0.00215), peaking at 2 h (657.45 ± 59.41 pg/ml, *P* = 0.00012), followed by temporary decline at 6 h (*P* = 0.414) and subsequent sustained increase to a maximum at 48 h (917.33 ± 62.29 pg/ml, *P* = 0.00217). No significant MCP-1 changes were observed in the sham group(*P* > 0.05).

Tissue remodeling markers TIMP-1 and CTGF showed sustained upward trends (Figures 2B,E). TIMP-1 increased from baseline (22.30 ± 3.12 ng/ml) at all time points (*P* = 0.013–0.522), peaking at 48 h (49.89 ± 2.40 ng/ml). Sham group TIMP-1 levels remained consistent. CTGF changes were more pronounced, rising significantly from baseline (955.6 ± 125.1 pg/ml) at 2 h (1,437.7 ± 120.3 pg/ml, *P* = 0.0036) and reaching 2,415.3 ± 75.9 pg/ml by 24 h (*P* = 0.0013). In the sham group, CTGF levels showed no significant deviation from baseline.

### 3.3 Serum biomarkers showing fluctuant elevation patterns

**Figure 3:**
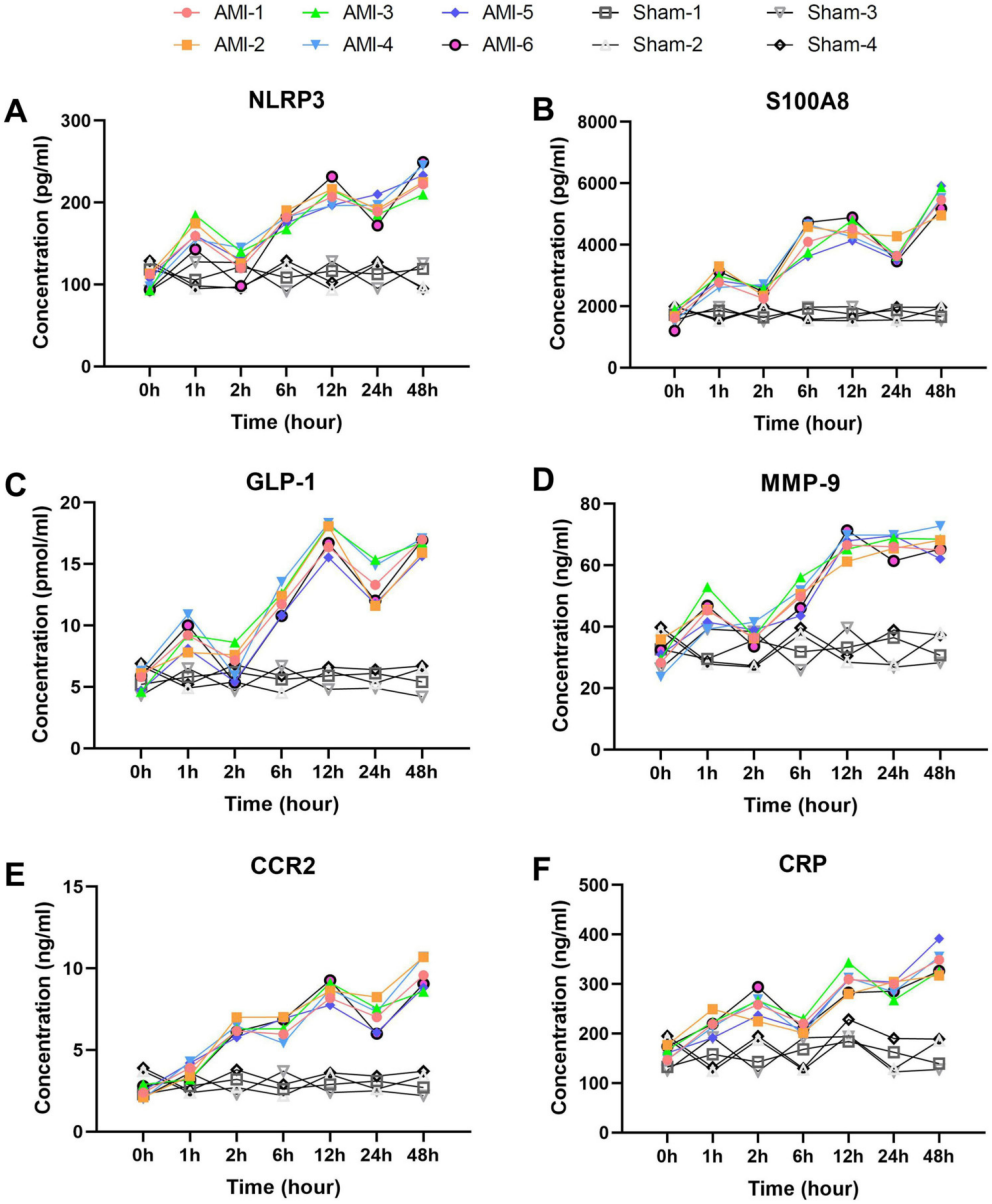
Early elevation with wavelike fluctuation patterns of inflammatory and chemotaxis-related biomarkers post-AMI. **(A)** NLRP3 showing characteristic fluctuant elevation with initial peak at 1 h (*P* = 0.00031 vs. baseline), temporary decline at 2 h (*P* = 0.0017), and secondary rise to 48 h (*P* = 0.0048). Sham group levels remained stable. **(B)** S100A8 demonstrating dramatic surge at 1 h (*P* = 0.00034) with similar fluctuant pattern. No significant changes in sham group. **(C)** GLP-1 exhibiting biphasic elevation from 1 h (*P* = 0.00042) to 48 h (*P* = 0.0015). Sham group showed no significant fluctuation. **(D)** MMP-9 displaying rapid rise to 12 h (*P* = 0.0015) despite temporary decline at 6 h (*P* = 0.0384). Levels remained unaltered in sham group. **(E)** CCR2 showing progressive increase from 2 h (*P* = 0.00037) to 48 h (*P* = 0.00061). Sham group maintained baseline levels. **(F)** CRP exhibiting continuous elevation to 48 h (*P* = 0.0039). No elevation observed in sham animals. Data are mean ± SEM. *n* = 6, ll *P* values represent comparisons with baseline measurements.

**Figure 4:**
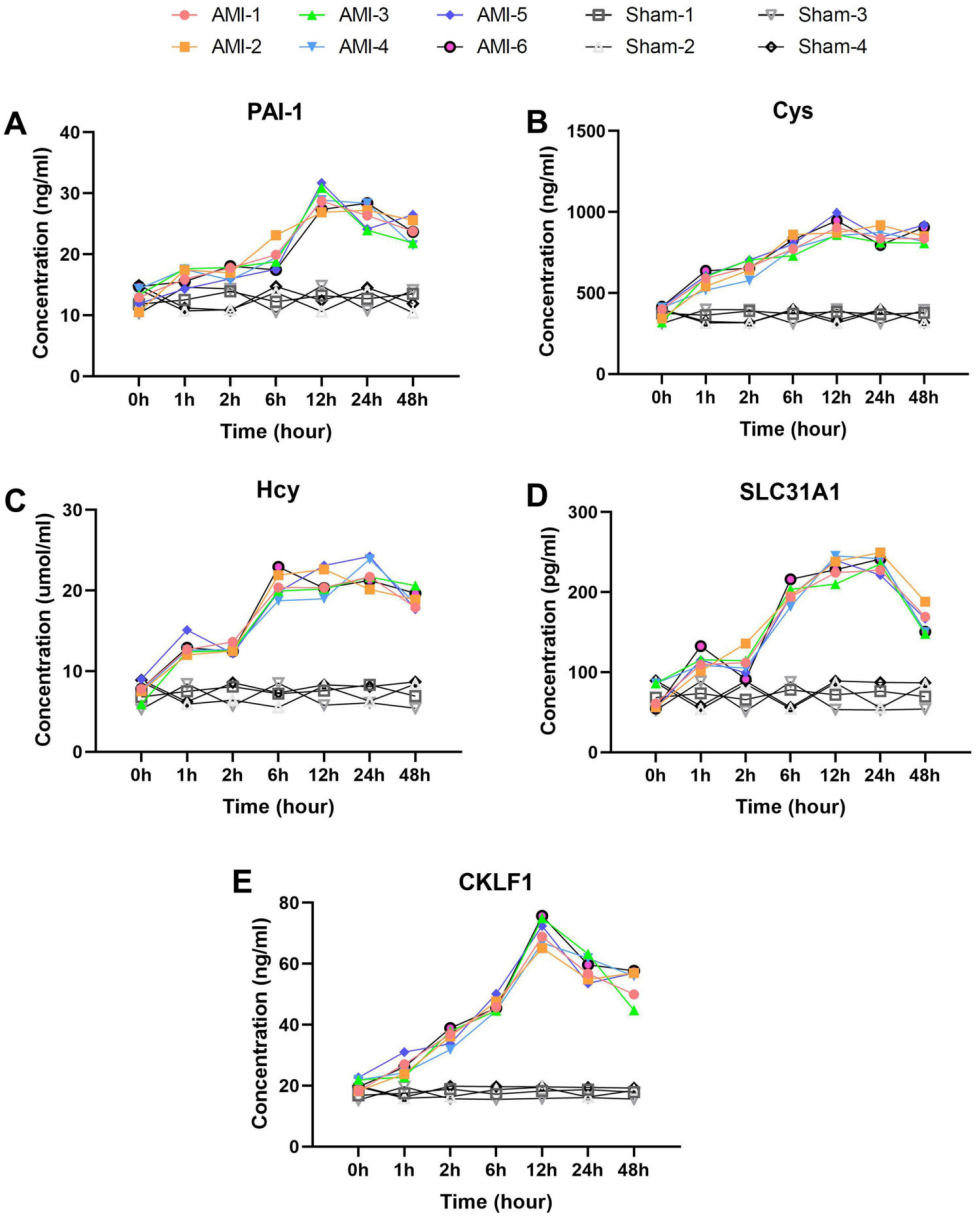
Delayed elevation with peak-and-decline patterns of fibrosis and metabolic biomarkers post-AMI. **(A)** PAI-1 showing continuous rise to 12 h (*P* = 0.0011) followed by slight decline. Sham group levels remained stable (*P* > 0.05). **(B)** Cys and **(C)** Hcy displaying parallel trends with peaks at 12 h and 24 h respectively (Hcy *P* = 0.00008). Both markers showed no significant changes in sham group. **(D)** SLC31A1 demonstrating sharp increase at 1 h (*P* = 0.00001), peak at 24 h, and significant decline by 48 h. No significant fluctuation was observed in sham group. **(E)** CKLF1 exhibiting peak elevation at 12 h before decline. Sham animals maintained baseline levels throughout. Data are mean ± SEM. *n* = 6, All *P* values represent comparisons with baseline measurements.

Among inflammatory markers, NLRP3 (Figure 3A) displayed characteristic fluctuant elevation: baseline 102.97 ± 9.35 pg/ml increased sharply at 1 h (162.63 ± 14.88 pg/ml, *P* = 0.00031), temporarily declined at 2 h (*P* = 0.0017), then rose continuously to peak at 48 h (230.82 ± 14.91 pg/ml, *P* = 0.0048). In contrast, sham group NLRP3 levels remained stable throughout. S100A8 (Figure 3B) showed more dramatic changes: baseline 1,623.20 ± 240.19 pg/ml surged at 1 h (2,940.48 ± 250.54 pg/ml, *P* = 0.00034), temporarily declined at 2 h, then peaked at 48 h (5,483.50 ± 379.69 pg/ml, *P* = 0.00079). No significant S100A8 changes occurred in sham group. CRP (Figure 3F) rose continuously from baseline (162.14 ± 12.90 pg/ml) to 258.21 ± 24.66 pg/ml at 2 h, then peaked at 48 h (344.19 ± 27.52 pg/ml, *P* = 0.0039). CKLF1 (Figure 4E) showed similar kinetics, peaking at 12 h (70.68 ± 4.33 ng/ml) before declining. CKLF1 levels in sham animals remained consistent.

Among chemotaxis-related markers, CCR2 (Figure 3E) increased progressively from baseline (2.43 ± 0.34 ng/ml) to 9.58 ± 0.94 ng/ml at 48 h (*P* = 0.00061), with significant elevation at 2 h (*P* = 0.00037). Sham group CCR2 levels exhibited minimal fluctuation. MMP-9 (Figure 3D) showed comparable kinetics: baseline 30.09 ± 4.11 ng/ml rose rapidly to 67.03 ± 3.60 ng/ml at 12 h (*P* = 0.0015), remaining elevated despite temporary decline at 6 h (*P* = 0.0384). MMP-9 values in sham group showed no significant change.

Metabolic/fibrosis markers exhibited unique patterns. GLP-1 (Figure 3C) showed biphasic elevation: baseline 5.6 ± 0.73 pmol/ml increased rapidly at 1 h (9.2 ± 1.14 pmol/ml, *P* = 0.00042), fluctuating upward to peak at 48 h (16.56 ± 0.62 pmol/ml, *P* = 0.0015). GLP-1 levels in sham group remained at baseline range. PAI-1 (Figure 4A) rose continuously from baseline (12.95 ± 1.56 ng/ml) to 29.07 ± 1.91 ng/ml at 12 h (*P* = 0.0011), then slightly declined. Sham group PAI-1 levels were unaltered. Cys and Hcy showed parallel trends (Figures 4B,C), with Cys peaking at 12 h (905.40 ± 55.87 ng/ml) and Hcy at 24 h (22.15 ± 1.59 μmol/L, *P* = 0.00008). Both markers remained stable in sham group. SLC31A1 increased sharply at 1 h (113.83 ± 10.53 pg/ml, *P* = 0.00001), peaked at 24 h (236.17 ± 10.33 pg/ml), then declined significantly by 48 h. No significant SLC31A1 changes were detected in sham group.

### 3.4 Serum biomarkers showing delayed elevation (2–6 h post-AMI)

**Figure 5:**
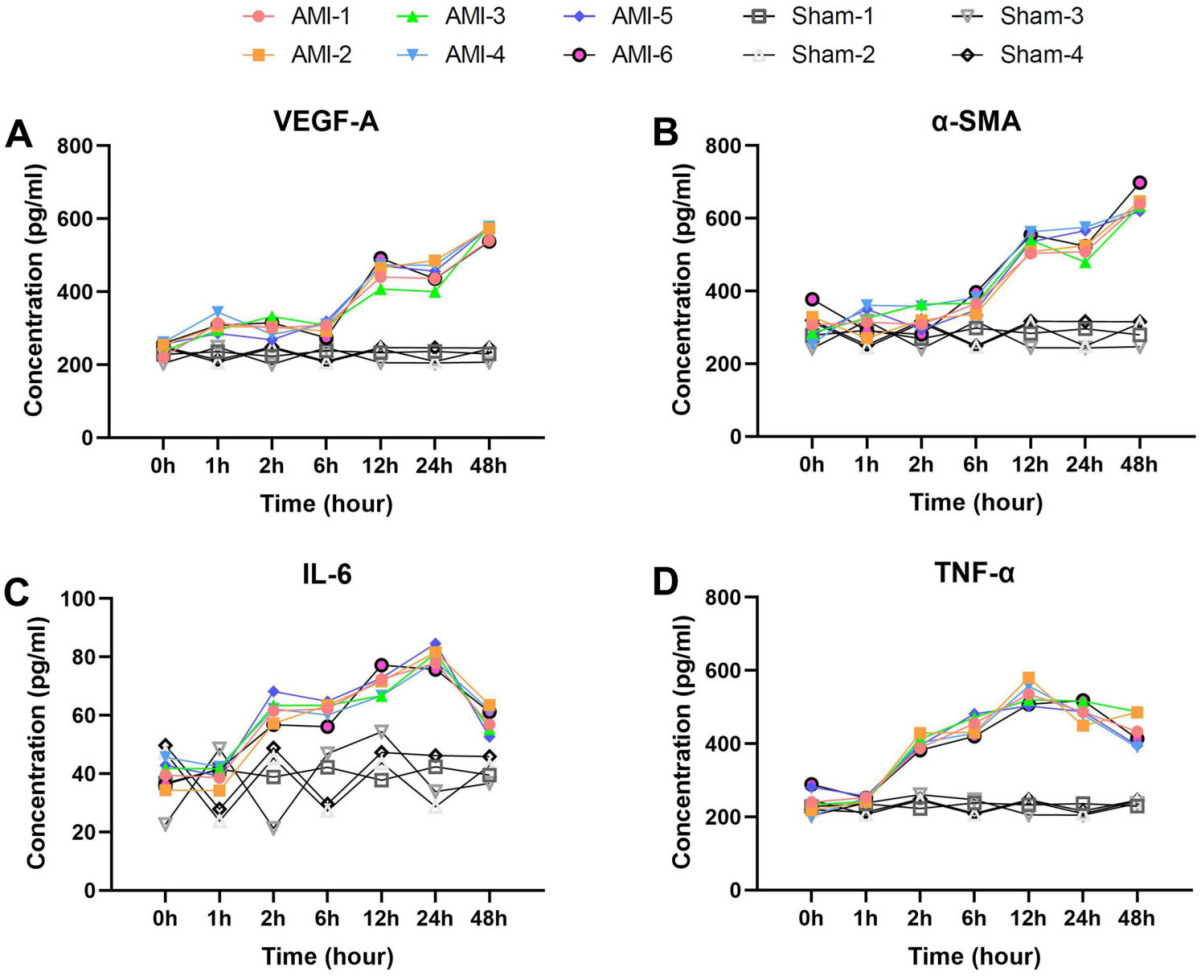
Delayed elevation patterns of vascular remodeling and inflammatory biomarkers post-AMI. **(A)** VEGF-A showing biphasic response (baseline 247.9 ± 16.0 pg/ml) with delayed elevation to 458.4 ± 29.8 pg/ml at 12 h (*P* = 0.0002), peaking at 564.5 ± 19.8 pg/ml (48 h). Sham group showed no significant changes. **(B)** α-SMA demonstrating delayed first increase to 366.2 ± 21.7 pg/ml at 6 h (*P* = 0.042), peaking at 644.5 ± 27.8 pg/ml (48 h). Levels remained stable in sham group. **(C)** IL-6 (baseline 40.1 ± 4.2 pg/ml) elevating to 61.6 ± 4.2 pg/ml at 2 h (*P* = 7.14 × 10^−5^), peaking at 79.9 ± 3.2 pg/ml (24 h). No significant elevation in sham group. **(D)** TNF-α showing rapid increase to 401.8 ± 17.2 pg/ml at 2 h (*P* = 1.57 × 10^−5^), peaking at 534.0 ± 30.2 pg/ml (12 h). Sham group maintained baseline levels. Data are mean ± SEM. All *P* values vs. baseline.

Here we analyzed four serum biomarkers closely associated with vascular remodeling and inflammatory responses. VEGF-A (Figure 5A) exhibited a typical biphasic elevation pattern, showing no significant increase from the baseline level of 247.87 ± 15.98 pg/ml to 308.76 ± 19.98 pg/ml within 1 h, remaining stable thereafter, then significantly increasing again to 458.41 ± 29.81 pg/ml at 12 h (*p* = 0.0002), and peaking at 564.54 ± 19.81 pg/ml by 48 h (*p* = 0.0003). In the sham group, VEGF-A levels remained stable throughout.

α-SMA (Figure 5B) demonstrated delayed elevation characteristics, with a baseline level of 305.40 ± 43.61 pg/ml, showing its first significant increase to 366.24 ± 21.72 pg/ml only at 6 h (*p* = 0.042), followed by a sharp rise to 534.29 ± 24.13 pg/ml at 12 h (*p* = 2.74 × 10^−6^), and reaching its peak at 644.53 ± 27.84 pg/ml by 48 h (*p* = 0.0027). No significant α-SMA changes were observed in the sham group.

The proinflammatory cytokines IL-6 and TNF-α both displayed characteristic changes (Figures 5C,D). IL-6 increased significantly from a baseline of 40.09 ± 4.19 pg/ml to 61.56 ± 4.22 pg/ml at 2 h (*p* = 7.14 × 10^−5^), peaked at 79.94 ± 3.19 pg/ml by 24 h (*p* = 0.0145), and then declined at 48 h (*p* = 0.0005). IL-6 levels in sham animals showed no notable variation. TNF-α showed more pronounced changes, increasing sharply from a baseline of 244.37 ± 34.32 pg/ml to 401.80 ± 17.20 pg/ml at 2 h (*p* = 1.57 × 10^−5^), peaking at 533.96 ± 30.21 pg/ml by 12 h (*p* = 0.0071), and remaining at relatively high levels at 48 h (*p* = 0.0498). TNF-α levels remained consistent in the sham group.

### 3.5 Other elevated serum biomarkers

**Figure 6:**
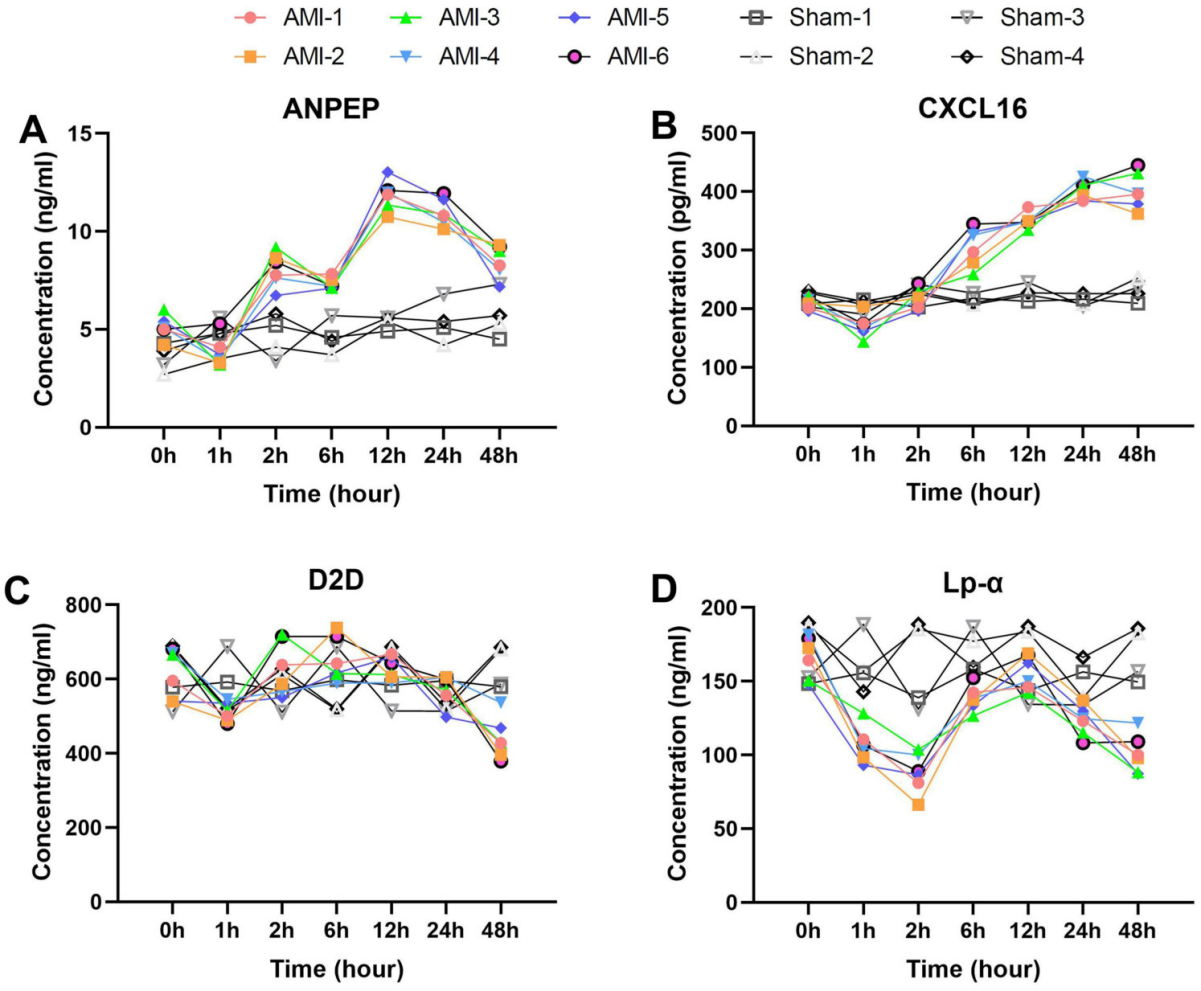
Unique temporal patterns of special biomarkers post-AMI. **(A)** ANPEP (baseline 5.1 ± 0.6 ng/ml) decreasing to 3.8 ± 0.8 ng/ml (1 h) then peaking at 11.8 ± 0.8 ng/ml (12 h). Sham group levels remained stable throughout (*P* > 0.05). **(B)** CXCL16 showing initial drop to 170.8 ± 19.6 pg/ml (1 h) before peaking at 401.8 ± 16.8 pg/ml (24 h). No significant changes observed in sham group. **(C)** D2D demonstrating dynamic changes from 615.6 ± 65.7 ng/ml (baseline) to peak 652.8 ± 60.1 ng/ml (6 h). Sham group maintained consistent baseline levels. **(D)** Lp-α decreasing to 106.9 ± 12.2 ng/ml (1 h) then rebounding to 156.3 ± 11.5 ng/ml (12 h). Levels remained unaltered in sham animals. Data are mean ± SEM. All *P* values vs. baseline unless noted.

We then analyzed the temporal change characteristics of two special biomarkers, ANPEP and CXCL16 (Figures 6A,B). ANPEP exhibited a unique fluctuating elevation pattern, decreasing significantly from baseline 5.1 ± 0.6 ng/ml to 3.8 ± 0.8 ng/ml at 1 h (*p* = 0.030), then rebounding sharply to 8.1 ± 0.9 ng/ml at 2 h (*p* = 0.0003). After a brief decline at 6 h, it reached its peak at 12 h (11.8 ± 0.8 ng/ml, *p* = 7.0 × 10^−5^), and decreased to 8.5 ± 0.8 ng/ml by 48 h (*p* = 0.0038). ANPEP levels in the sham group remained stable across all time points.

CXCL16 showed a progressively delayed elevation characteristic, decreasing significantly from baseline 210.9 ± 10.6 pg/ml to 170.8 ± 19.6 pg/ml at 1 h (*p* = 0.0097), then returning to baseline levels at 2 h (*p* = 0.0073). It continued to rise thereafter, reaching 306.1 ± 33.4 pg/ml at 6 h (*p* = 0.0023), peaking at 401.8 ± 16.8 pg/ml by 24 h (*p* = 0.0046), and maintaining a plateau phase at 48 h (*p* = 0.997). No significant CXCL16 fluctuations were detected in the sham group.

### 3.6 Serum biomarkers showing post-AMI decrease

Finally, we present two biomarkers that showed overall decreases post-operation.

Serum D2D levels exhibited a unique dynamic pattern after AMI (Figure 6C). The baseline level was 615.60 ± 65.68 ng/ml, which decreased significantly to 510.93 ± 25.77 ng/ml at 1 h post-AMI (*p* = 0.015). It then rebounded to 629.55 ± 74.31 ng/ml at 2 h (*p* = 0.025) and peaked at 652.77 ± 60.06 pg/ml by 6 h (compared to 2 h, *p* = 0.531). After 12 h, it began to decline gradually, further decreasing to 439.63 ± 56.71 ng/ml by 48 h. In contrast, D2D levels in the sham group remained stable throughout the observation period.

Serum Lp-α levels showed significant time-dependent changes after AMI (Figure 6D). The baseline level was 165.99 ± 14.31 ng/ml, which rapidly decreased to 106.88 ± 12.20 ng/ml at 1 h post-AMI (*p* = 0.0009). This downward trend continued, reaching the lowest value of 87.76 ± 13.51 ng/ml at 2 h. Subsequently, it showed a significant rebound to 138.45 ± 8.63 ng/ml at 6 h (*p* = 0.0010) and peaked at 156.25 ± 11.52 ng/ml by 12 h (compared to 6 h, *p* = 0.0091). In the later phase (24–48 h), it exhibited a downward trend again, decreasing to 123.00 ± 10.24 ng/ml at 24 h and further declining to 100.71 ± 13.09 ng/ml by 48 h. No significant Lp-α fluctuations were observed in the sham group, with levels maintaining near baseline values.

## 4 Discussion

Through systematic analysis of dynamic changes in 25 serum biomarkers within 48 h after AMI, this study has revealed the temporal characteristics of pathophysiological processes including myocardial injury, inflammatory response, and tissue remodeling. More importantly, it provides new experimental evidence for early AMI diagnosis and mechanistic research. The results demonstrate that different categories of biomarkers exhibit distinct dynamic patterns, which not only reflect the complex pathophysiological processes post-AMI but also offer crucial clues for optimizing clinical diagnosis and therapeutic strategies.

Regarding early diagnostic biomarkers, this study identified multiple biomarkers showing significant elevation within 1–6 h after AMI onset. hs-TnI demonstrated statistically significant elevation as early as 2 h, supporting the clinical value of high-sensitivity troponin testing recently promoted in clinical practice. Notably, certain inflammatory markers like IL-1β and S100A8 showed rapid elevation within 1 h, even earlier than hs-TnI. These ultra-early changes suggest their potential involvement in immediate ischemia-induced inflammatory responses. Meanwhile, early elevation of MMP-9 and PAI-1 reflects acute activation of the coagulation-fibrinolysis system (17, 18), while the biphasic pattern of GLP-1 implies the regulatory role of the gut-brain axis during myocardial ischemic stress (19, 20). Combined detection of these biomarkers may further advance the diagnostic window for AMI, creating more favorable conditions for early clinical intervention.

From a pathophysiological perspective, the inflammatory response post-AMI exhibited distinct phase-specific characteristics. The early phase (0–2 h) was marked by rapid release of mediators like IL-1β and S100A8; the intermediate phase (6–12 h) was accompanied by significant elevation of pro-inflammatory factors such as TNF-α and IL-6; while the late phase (24–48 h) was dominated by sustained expression of chemokines including MCP-1 and CXCL16. This temporal progression closely aligns with the pathological process of ischemia-reperfusion injury, reflecting the natural disease course from acute injury to chronic inflammation (21, 22). In terms of tissue remodeling, the progressive elevation of CTGF (23) and TIMP-1 (24) corresponded with myocardial fibrosis progression, while the delayed elevation of α-SMA suggested that myofibroblast activation might be a secondary process following inflammatory responses (25).

Particularly noteworthy are the changing patterns of several special biomarkers. The unique pattern of Lp-α post-AMI, characterized by early significant decrease followed by transient recovery, may be related to acute-phase inhibition of hepatic synthesis and associated with gut microbiota—a mechanism distinctly different from traditional lipid metabolism markers (26). The sustained downregulation of D2D might reflect protective inhibition of dopaminergic neurons, and the correlation between this change and cardiac function deterioration warrants further investigation (27–29). Additionally, the delayed elevation of CXCL16 (30) suggests its primary involvement in chronic inflammatory processes rather than acute injury responses, while the changing pattern of copper transporter protein SLC31A1 (31) provides new experimental evidence for the role of metal ion metabolism in AMI.

The findings of this study hold significant clinical translational value. Firstly, We found that five biomarkers (IL-1β, S100A8, BNP, SLC31A1 and Cys) showed an increase of over 70% at 1 h post-AMI, the combined detection strategy based on these biomarkers could significantly improve ultra-early diagnosis rates for AMI. Secondly, the temporal characteristics of different biomarker categories provide clear therapeutic windows—for instance, interventions targeting early inflammatory responses (0–6 h) and late fibrosis (12–24 h) may require different strategies. Furthermore, the unique changing patterns of biomarkers like GLP-1 and D2D suggest that neuroendocrine regulation may become a new therapeutic target. These findings provide important theoretical foundations for precise diagnosis and personalized treatment of AMI.

Nevertheless, this study has some limitations. Firstly, results from animal models require further clinical validation. Secondly, the study primarily focused on changes within 48 h, and longer-term dynamic patterns need further elucidation. Thirdly, the exclusive use of young adult male rats may limit the clinical translatability of our findings. The absence of aged rats, which better simulate the elderly human population most susceptible to AMI, and the lack of female rats, which prevents the exploration of potential sex-specific biomarker responses, constrain the generalizability of our results. Furthermore, the deliberate omission of postoperative antibiotic prophylaxis, while intended to avoid confounding the natural inflammatory response, may have introduced infection as an unmeasured confounding variable. Additionally, the interaction mechanisms among different biomarkers require more in-depth research. Future studies could employ transcriptomics and proteomics approaches to reveal the complex molecular network regulatory mechanisms post-AMI from a more systematic perspective. Meanwhile, prospective clinical studies could be conducted based on the promising biomarker combinations identified in this study to evaluate their practical application value in early AMI diagnosis and prognosis prediction.

In conclusion, through systematic analysis of dynamic serum biomarker profiles post-AMI, this study has not only identified multiple promising early diagnostic biomarkers but also revealed the temporal characteristics of different pathophysiological processes, providing important experimental evidence for understanding AMI pathogenesis and developing new diagnostic and therapeutic strategies. These findings will facilitate the advancement of early diagnosis and precision treatment for AMI, ultimately improving clinical outcomes for patients.

## Data Availability

The original contributions presented in the study are included in the article/Supplementary Material, further inquiries can be directed to the corresponding authors.

## References

[1] Roth GA Mensah GA Johnson CO Addolorato G Ammirati E Baddour LM Global burden of cardiovascular diseases and risk factors, 1990–2019: update from the Gbd 2019 study. J Am Coll Cardiol. (2020) 76(25):2982–3021. 10.1016/j.jacc.2020.11.010 33309175 PMC7755038

[2] Aslanger EK Yıldırımtürk Ö Şimşek B Bozbeyoğlu E Şimşek MA Yücel Karabay C Diagnostic accuracy of electrocardiogram for acute coronary occlusion resulting in myocardial infarction (difoccult study). Int J Cardiol Heart Vasc. (2020) 30:100603. 10.1016/j.ijcha.2020.100603 32775606 PMC7399112

[3] Wiebe J Nef HM Hamm CW. Current status of bioresorbable scaffolds in the treatment of coronary artery disease. J Am Coll Cardiol. (2014) 64(23):2541–51. 10.1016/j.jacc.2014.09.041 25500240

[4] Bezati S Ventoulis I Bistola V Verras C Matsiras D Polyzogopoulou E Copeptin in acute myocardial infarction: is there a role in the era of high-sensitivity troponins? J Cardiovasc Dev Dis. (2025) 12(4):144. 10.3390/jcdd12040144 40278203 PMC12027642

[5] Arai M Asaumi Y Honda S Ogata S Kiyoshige E Nakao K Association between left ventricular reverse remodelling and the B-type natriuretic Peptide-Cgmp cascade after anterior acute myocardial infarction. Open Heart. (2025) 12(1):e002927. 10.1136/openhrt-2024-002927 39800436 PMC11751991

[6] Couch LS Garrard JW Henry JA Kotronias RA Alaour B De Maria GL Comparison of troponin and natriuretic peptides in takotsubo syndrome and acute coronary syndrome: a meta-analysis. Open Heart. (2024) 11(1):e002607. 10.1136/openhrt-2024-002607 38508657 PMC10952941

[7] Duque-Ossa LC García-Ferrera B Reyes-Retana JA. Troponin I as a biomarker for early detection of acute myocardial infarction. Curr Probl Cardiol. (2023) 48(5):101067. 10.1016/j.cpcardiol.2021.101067 34826431

[8] Alpert JS Thygesen K Antman E Bassand JP. Myocardial infarction redefined–a consensus document of the joint European society of cardiology/American college of cardiology committee for the redefinition of myocardial infarction. J Am Coll Cardiol. (2000) 36(3):959–69. 10.1016/S0735-1097(00)00804-4 10987628

[9] Yang Z Min Zhou D. Cardiac markers and their point-of-care testing for diagnosis of acute myocardial infarction. Clin Biochem. (2006) 39(8):771–80. 10.1016/j.clinbiochem.2006.05.011 16836992

[10] Cai S Zhao M Zhou B Yoshii A Bugg D Villet O Mitochondrial dysfunction in macrophages promotes inflammation and suppresses repair after myocardial infarction. J Clin Invest. (2023) 133(4):e159498. 10.1172/JCI159498 36480284 PMC9927948

[11] Neri M Fineschi V Di Paolo M Pomara C Riezzo I Turillazzi E Cardiac oxidative stress and inflammatory cytokines response after myocardial infarction. Curr Vasc Pharmacol. (2015) 13(1):26–36. 10.2174/15701611113119990003 23628007

[12] Wu X Reboll MR Korf-Klingebiel M Wollert KC. Angiogenesis after acute myocardial infarction. Cardiovasc Res. (2021) 117(5):1257–73. 10.1093/cvr/cvaa287 33063086

[13] Oprescu N Micheu MM Scafa-Udriste A Popa-Fotea N-M Dorobantu M. Inflammatory markers in acute myocardial infarction and the correlation with the severity of coronary heart disease. Ann Med. (2021) 53(1):1041–7. 10.1080/07853890.2021.1916070 34180324 PMC8245096

[14] Eggers KM Lindhagen L Lindhagen L Baron T Erlinge D Hjort M Predicting outcome in acute myocardial infarction: an analysis investigating 175 circulating biomarkers. Eur Heart J Acute Cardiovasc Care. (2021) 10(7):806–12. 10.1093/ehjacc/zuaa014 34100060

[15] Goldman S Raya TE. Rat infarct model of myocardial infarction and heart failure. J Card Fail. (1995) 1(2):169–77. 10.1016/1071-9164(95)90019-5 9420647

[16] Ai X Yan B Witman N Gong Y Yang L Tan Y Transient secretion of vegf protein from transplanted Hipsc-Cms enhances engraftment and improves rat heart function post mi. Mol Ther. (2022) 31(1):211–29. 10.1016/j.ymthe.2022.08.012 35982619 PMC9840120

[17] Krychtiuk KA Speidl WS Giannitsis E Gigante B Gorog DA Jaffe AS Biomarkers of coagulation and fibrinolysis in acute myocardial infarction: a joint position paper of the association for acute cardiovascular care and the European society of cardiology working group on thrombosis. Eur Heart J Acute Cardiovasc Care. (2021) 10(3):343–55. 10.1093/ehjacc/zuaa025 33620437

[18] Liberale L Puspitasari YM Ministrini S Akhmedov A Kraler S Bonetti NR Jcad promotes arterial thrombosis through Pi3k/Akt modulation: a translational study. Eur Heart J. (2023) 44(20):1818–33. 10.1093/eurheartj/ehac641 36469488 PMC10200023

[19] Sattar N Lee MMY Kristensen SL Branch KRH Del Prato S Khurmi NS Cardiovascular, mortality, and kidney outcomes with Glp-1 receptor agonists in patients with type 2 diabetes: a systematic review and meta-analysis of randomised trials. Lancet Diabetes Endocrinol. (2021) 9(10):653–62. 10.1016/S2213-8587(21)00203-5 34425083

[20] Boshchenko AA Maslov LN Mukhomedzyanov AV Zhuravleva OA Slidnevskaya AS Naryzhnaya NV Peptides are cardioprotective drugs of the future: the receptor and signaling mechanisms of the cardioprotective effect of glucagon-like peptide-1 receptor agonists. Int J Mol Sci. (2024) 25(9):4900. 10.3390/ijms25094900 38732142 PMC11084666

[21] Welt FGP Batchelor W Spears JR Penna C Pagliaro P Ibanez B Reperfusion injury in patients with acute myocardial infarction: Jacc scientific statement. J Am Coll Cardiol. (2024) 83(22):2196–213. 10.1016/j.jacc.2024.02.056 38811097

[22] Francisco J Del Re DP. Inflammation in myocardial ischemia/reperfusion injury: underlying mechanisms and therapeutic potential. Antioxidants (Basel). (2023) 12(11):1944. 10.3390/antiox12111944 38001797 PMC10669026

[23] Vainio LE Szabó Z Lin R Ulvila J Yrjölä R Alakoski T Connective tissue growth factor inhibition enhances cardiac repair and limits fibrosis after myocardial infarction. JACC Basic Transl Sci. (2019) 4(1):83–94. 10.1016/j.jacbts.2018.10.007 30847422 PMC6390503

[24] Cavusoglu E Ruwende C Chopra V Yanamadala S Eng C Clark LT Tissue inhibitor of metalloproteinase-1 (Timp-1) is an independent predictor of all-cause mortality, cardiac mortality, and myocardial infarction. Am Heart J. (2006) 151(5):1101.e1–e8. 10.1016/j.ahj.2006.02.029 16644343

[25] Cho DI Ahn MJ Cho HH Cho M Jun JH Kang BG Angptl4 stabilizes atherosclerotic plaques and modulates the phenotypic transition of vascular smooth muscle cells through Klf4 downregulation. Exp Mol Med. (2023) 55(2):426–42. 10.1038/s12276-023-00937-x 36782020 PMC9981608

[26] Zuo J Wang P Xue K Tan Y Zhang T Li Y Lipid alterations in acute myocardial infarction are associated with gut microbiota. Microbiol Spectr. (2025) 13(4):e0237024. 10.1128/spectrum.02370-24 39969201 PMC11960122

[27] Manrique-Maldonado G Altamirano-Espinoza AH Rivera-Mancilla E Hernández-Abreu O Villalón CM. Activation of dopamine D3 receptor subtypes inhibits the neurogenic systemic vasodilation induced by stimulation of the perivascular cgrpergic discharge. ACS Chem Neurosci. (2019) 10(8):3751–7. 10.1021/acschemneuro.9b00277 31343160

[28] Teisman AC van Veldhuisen DJ Scholtens E Maselbas W van Gilst WH. Effects of selective dopaminergic receptor stimulation on ventricular remodeling after experimental myocardial infarction in rats. J Card Fail. (1997) 3(3):199–205. 10.1016/S1071-9164(97)90016-6 9330128

[29] Lazou A Markou T Zioga M Vasara E Efstathiou A Gaitanaki C. Dopamine mimics the cardioprotective effect of ischemic preconditioning via activation of Alpha1-adrenoceptors in the isolated rat heart. Physiol Res. (2006) 55(1):1–8. 10.33549/physiolres.930694 15857158

[30] Lehrke M Millington SC Lefterova M Cumaranatunge RG Szapary P Wilensky R Cxcl16 is a marker of inflammation, atherosclerosis, and acute coronary syndromes in humans. J Am Coll Cardiol. (2007) 49(4):442–9. 10.1016/j.jacc.2006.09.034 17258089

[31] Zhou S Wang L Huang X Wang T Tang Y Liu Y Comprehensive bioinformatics analytics and *in vivo* validation reveal Slc31a1 as an emerging diagnostic biomarker for acute myocardial infarction. Aging (Albany NY). (2024) 16(9):8361–77. 10.18632/aging.205199 38713173 PMC11132003

